# β-N-methylamino-L-alanine (BMAA) suppresses cell cycle progression of non-neuronal cells

**DOI:** 10.1038/s41598-018-36418-9

**Published:** 2018-12-20

**Authors:** Saki Okamoto, Shigeyuki Esumi, Kayoko Hamaguchi-Hamada, Shun Hamada

**Affiliations:** 1 Department of Food and Health Sciences, International College of Arts and Sciences, Fukuoka Women’s University, 1-1-1 Kasumigaoka, Higashi-ku, Fukuoka 813-8529 Japan; 2 Department of Morphological Neural Science, Graduate School of Medical Sciences, Kumamoto University, 1-1-1 Honjo, Chuo-ku, Kumamoto 860-8556 Japan; 3 Department of Food Sciences and Nutrition, School of Human Environmental Sciences, Mukogawa Women’s University, 6-46 Ikebiraki, Nishinomiya, Hyogo 663-8558 Japan

## Abstract

β-N-methylamino-L-alanine (BMAA), a natural non-proteinaceous amino acid, is a neurotoxin produced by a wide range of cyanobacteria living in various environments. BMAA is a candidate environmental risk factor for neurodegenerative diseases such as amyotrophic lateral sclerosis and Parkinson-dementia complex. Although BMAA is known to exhibit weak neuronal excitotoxicity via glutamate receptors, the underlying mechanism of toxicity has yet to be fully elucidated. To examine the glutamate receptor-independent toxicity of BMAA, we investigated the effects of BMAA in non-neuronal cell lines. BMAA potently suppressed the cell cycle progression of NIH3T3 cells at the G1/S checkpoint without inducing plasma membrane damage, apoptosis, or overproduction of reactive oxygen species, which were previously reported for neurons and neuroblastoma cells treated with BMAA. We found no evidence that activation of glutamate receptors was involved in the suppression of the G1/S transition by BMAA. Our results indicate that BMAA affects cellular functions, such as the division of non-neuronal cells, through glutamate receptor-independent mechanisms.

## Introduction

β-N-methylamino-L-alanine (BMAA), a natural non-proteinaceous amino acid, is a neurotoxin^1–8^ produced by a wide range of cyanobacteria living in various environments^9^. BMAA becomes concentrated through the food chain^10,11^, and high concentrations of BMAA have been detected in aquatic animals at high trophic levels, such as mussels, oysters, and fish from the Baltic Sea^11^, a lagoon in southern France^12^, and a lake in New Hampshire^13^. BMAA is therefore a potential threat to human health in various locations.

BMAA was originally a proposed environmental risk factor for endemic neurodegenerative diseases, such as Parkinson-dementia complex (PDC) and amyotrophic lateral sclerosis (ALS), in the indigenous people of Guam^14^. This endemic disease is collectively called ALS/PDC due to the potential link between ALS and PDC. According to the BMAA hypothesis^10,15^, BMAA is concentrated in the traditional foods of the indigenous people, gradually accumulates in the brain, and causes ALS/PDC with long latency. Moreover, sporadic ALS outside of Guam may be related to environmental BMAA exposure^12,16^.

One limitation of the BMAA hypothesis is that the underlying mechanism of toxicity has yet to be fully elucidated. BMAA is structurally related to another non-proteinaceous amino acid, β-N-oxalylamino-L-alanine (BOAA), which exhibits excitotoxicity and causes neurolathyrism^17^, a form of motor neuron disease induced by excessive ingestion of certain legumes. BMAA is excitotoxic against neurons through several types of glutamate receptors, including NMDA^5,7^, AMPA/kainite^4^, and mGluR5^18^. Intriguingly, the excitotoxicity of BMAA is strongly dependent on the presence of physiological concentrations of bicarbonate, and may be mediated by a carbamate adduct formed from the interaction of BMAA with bicarbonate^7,19^. However, the excitotoxicity of BMAA is markedly weaker than that of BOAA and glutamate^20^. Furthermore, a low concentration of BMAA that was not thought to be excitotoxic induced toxicity in a neuroblastoma cell line^21^. These findings suggest that BMAA has glutamate receptor-independent toxicity mechanisms.

Previous studies showed that BMAA is misincorporated into cellular proteins^21–23^, which may lead to adverse effects in cells^21,22^. Okle *et al*.^21^ reported that BMAA induced endoplasmic reticulum (ER) stress in a neuroblastoma cell line, possibly through mechanisms involving BMAA-associated proteins. Another group^22^ demonstrated that BMAA was misincorporated into proteins in place of L-serine in non-neuronal and neuroblastoma cells. In non-neuronal cells, BMAA induced intracellular autofluorescent bodies, which are indicative of protein aggregation^22^. These findings suggest that both non-neuronal cells and neurons are affected by BMAA through glutamate receptor-independent mechanisms.

To gain insight into BMAA toxicity, the effects of BMAA on the cellular function of non-neuronal cells and neurons need to be determined in an unbiased manner. To date, only a few studies^24,25^ have focused on BMAA toxicity in non-neuronal cells, specifically olfactory ensheathing (OE) cells, which are glial cells that wrap around the olfactory nerves. BMAA induced cell membrane damage in OE cells by markedly increasing the production of reactive oxygen species (ROS), and significantly enhancing mitochondrial activity and Ca^2+^ influx^24^. However, because glial cells express glutamate receptors, it is unclear whether these adverse effects of BMAA on OE cells occur independently of the activation of glutamate receptors^26^.

To examine whether BMAA affects cellular function in an unbiased manner, we investigated the effects of BMAA on a mouse fibroblast cell line, NIH3T3. We demonstrated that BMAA potently suppresses the cell cycle progression of NIH3T3 cells via glutamate receptor-independent mechanisms without inducing cell membrane damage, apoptosis, or overproduction of ROS.

## Results

**Figure 1:**
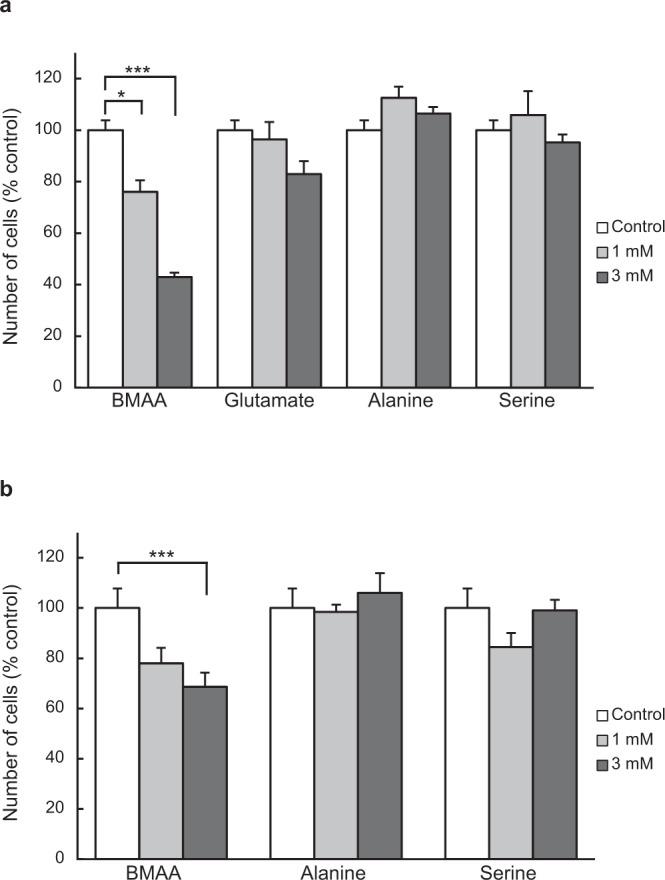
BMAA suppressed the proliferation of NIH3T3 cells (**a**) and HEK293T cells (**b**). Cells were cultured for 48 h in medium containing 1 or 3 mM BMAA, glutamate, alanine, or serine. Data were normalized to vehicle control values (open bars). *P < 0.05. ***P < 0.001. Error bars represent SEM, n = 5.

To investigate the effects of BMAA on non-neuronal cells, we initially analyzed the effects of BMAA on NIH3T3 cells, a fibroblast cell line. Incubation in medium containing 1 or 3 mM BMAA significantly suppressed increases in NIH3T3 cell number to a greater extent than in control cultures treated with vehicle (Fig. 1a). In contrast, glutamate, alanine, and serine did not suppress the increase in NIH3T3 cell number. To clarify whether increases in other non-neuronal cells were sensitive to BMAA, we conducted these experiments in HEK293T cells. We found that BMAA also suppressed increases in HEK293T cell number; however, the degree of suppression was less than that observed for NIH3T3 cells (Fig. 1b).

One possible cause of the suppressed proliferation of BMAA-treated cells is cell death induced by BMAA toxicity. To examine this possibility, we used a lactate dehydrogenase (LDH) assay to quantify LDH leakage into the culture supernatant from dying or dead cells. LDH leakage in the culture supernatants of NIH3T3 cells after BMAA treatment was similar to that from control cells treated with vehicle, glutamate, or alanine (Table 1). Therefore, BMAA did not induce cell death by compromising NIH3T3 cell membranes.

**Table 1:** BMAA had negligible effects on the cellular integrity of NIH3T3 cells.

Treatment		% LDH activity ± SEM
Triton X-100		100.00 ± 1.570
Control		2.44 ± 0.060
BMAA	1 mM	2.26 ± 0.040
	3 mM	2.86 ± 0.169
Glutamate	1 mM	2.16 ± 0.087
	3 mM	2.90 ± 0.247
Alanine	1 mM	2.04 ± 0.075
	3 mM	1.44 ± 0.075***
Serine	1 mM	1.98 ± 0.037
	3 mM	1.94 ± 0.103

We examined LDH leakage from cells treated with BMAA, glutamate, alanine, or serine for 48 h. Results are expressed as a ratio of the control, in which all cells were lysed with 1.5% Triton X-100. ***P < 0.001 significantly different from the vehicle control (n = 5).

BMAA induces apoptosis of neurons and neuroblastoma cells^1,21,22,27^. To detect apoptosis of BMAA-treated cells, we examined the activation of the caspase cascade using anti-active caspase-3 immunostaining. We found little to no active caspase-3-positive profiles with chromatin condensation among BMAA-treated cells or control cells (Table 2).

**Table 2:** BMAA did not activate the caspase cascade in NIH3T3 cells.

Treatment duration	Reagent	Nuclear profile	Active caspase-3-positive cells	%
48 h	Control	2939	4	0.14
48 h	BMAA	2930	2	0.07
48 h	Alanine	2345	0	0
24 h	Etoposide	1490	191	12.8

After cells were cultured in medium containing 1 mM BMAA or alanine for 48 h, we examined active caspase-3 immunoreactive cells with chromatin condensation (active caspase-3-positive cells). Control cells were treated with vehicle. We used 50 μM etoposide, an inducer of apoptosis, as a positive control. Nuclear profiles were the total number of nuclei examined by DAPI staining in each experiment.

**Figure 2:**
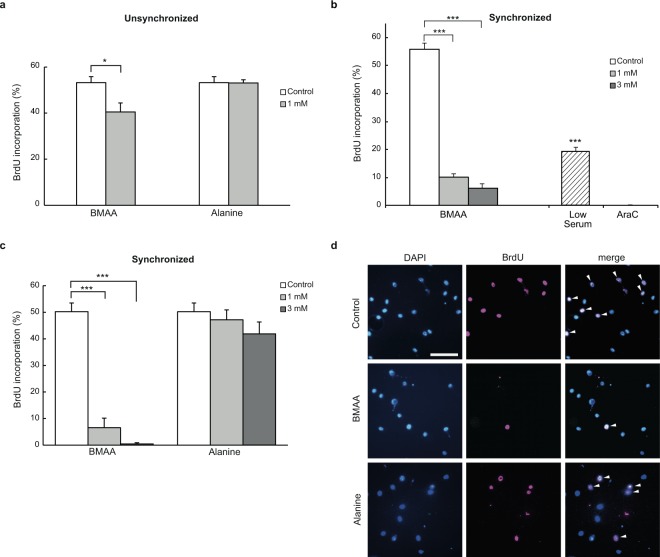
BMAA suppressed the cell cycle progression of NIH3T3 cells. (**a**) After cells were cultured in medium containing BMAA, alanine, or vehicle (open bars) for 48 h, they were labeled with BrdU for 2 h. Each bar represents the percentage of cells that incorporated BrdU. *P < 0.05. Error bars represent SEM, n = 3. (**b**) After G1-synchronized cells were cultured in DMEM with 10% FBS containing BMAA, 5 μM cytosine arabinoside (AraC, a cell cycle inhibitor) or vehicle (open bar), or with 0.5% FBS (Low Serum, a positive control for cell cycle arrest) for 16 h, they were labeled with BrdU for 2 h. Incorporation of BrdU was not observed in AraC-treated cells. ***P < 0.001 significantly different from the control. Error bars represent SEM, n = 3. (**c**) After G1-synchronized cells were cultured in medium containing BMAA, alanine or vehicle (open bars) for 16 h, cells were labeled with BrdU for 2 h. ***P < 0.001. Error bars represent SEM, n = 3. (**d**) Representative images from the experiment in (**c**). Arrowheads indicate BrdU-labeled cells. Scale = 50 μm.

The absence of apparent cell death of BMAA-treated cells prompted us to investigate whether BMAA suppresses cell cycle progression. To examine cell cycle progression in NIH3T3 cells treated with BMAA, we labeled cells with 5-bromo-2′-deoxyuridine (BrdU) for 2 h in medium containing BMAA. The ratio of BrdU-positive nuclei to DAPI-stained nuclei (the BrdU labeling index) was significantly lower for BMAA-treated cells than vehicle-treated control cells (Fig. 2a), suggesting that BMAA suppressed cell cycle progression. To identify which checkpoints were affected by BMAA, the cell cycle was synchronized at the G1 phase by serum starvation and cells were then cultured in normal medium with BMAA. BMAA markedly suppressed the G1/S transition (Fig. 2b); the BrdU labeling index for cells treated with 1 and 3 mM BMAA was 10.1% and 5.7%, respectively, whereas that for control cells was 56.2%. Furthermore, the BrdU labeling index for alanine-treated cells was similar to that for control cells (Fig. 2c,d).

**Figure 3:**
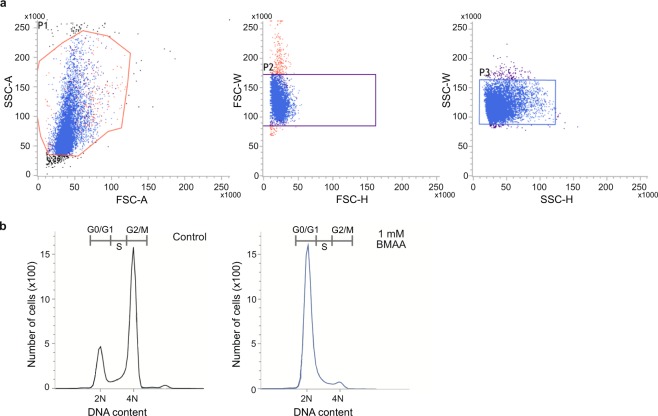
Flow cytometric analysis of NIH3T3 cells treated with BMAA. (**a**) Blue dots indicate cells that passed through three (SSC-A/FSC-A, FSC-W/FSC-H, and SSC-W/SSC-H) gates to eliminate doublet cells. (**b**) DNA was analyzed after G1-synchronized cells were cultured in medium containing 1 mM BMAA or vehicle for 22 h.

We also analyzed the cell cycle progression of BMAA-treated cells using flow cytometry (Fig. 3). After synchronized NIH3T3 cells were cultured with BMAA or vehicle, the DNA content of cells was examined when the majority of control cells entered the G2/M phase. In contrast to control cells, which mostly had signals corresponding to the G2/M phase, the majority of BMAA-treated cells had DNA signals corresponding to the G1/G0 phase (Fig. 3b).

**Figure 4:**
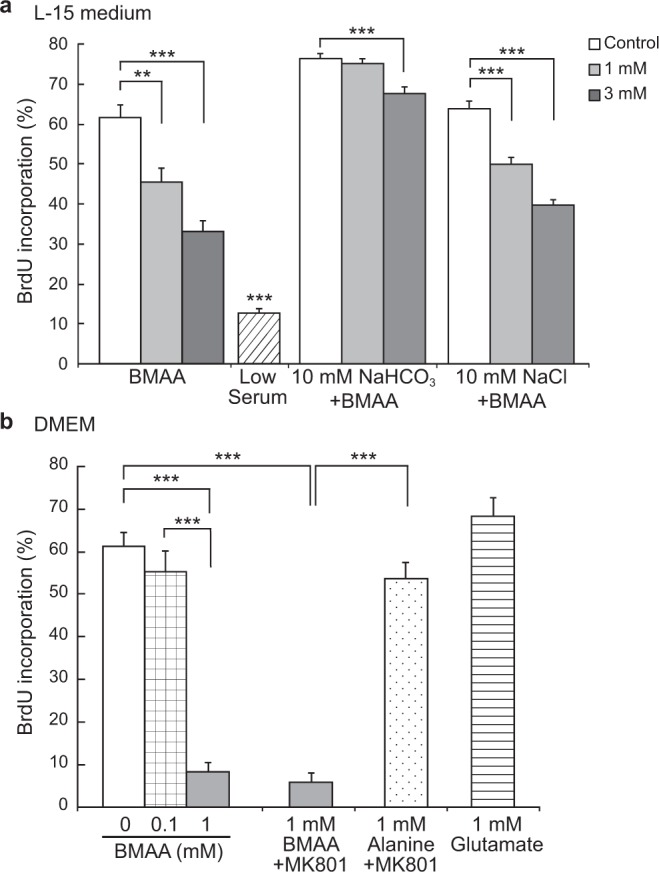
BMAA suppressed the G1/S transition of NIH3T3 cells without activating glutamate receptors. (**a**) After G1-synchronized cells were cultured in bicarbonate-free L-15 medium with 10% FBS containing BMAA or vehicle (open bar), 0.5% FBS (Low Serum, a positive control for cell cycle arrest) for 16 h, they were labeled with BrdU for 2 h. To examine whether the addition of bicarbonate to L-15 medium enhances the BMAA-mediated suppression of the G1/S transition, G1-synchronized cells were cultured in L-15 medium with 10% FBS containing BMAA and bicarbonate (10 mM NaHCO3 + BMAA) or BMAA and NaCl (10 mM NaCl + BMAA) as a negative control. Error bars represent SEM, n = 3. *P < 0.05. ***P < 0.001 significantly different from the vehicle control value. (**b**) After G1-synchronized cells were cultured in DMEM containing BMAA, BMAA with 50 μM MK-801, alanine with 50 μM MK-801, glutamate or vehicle (open bar) for 16 h, they were labeled with BrdU for 2 h. Error bars represent SEM, n = 3. ***P < 0.001.

We then examined whether the BMAA-mediated suppression of the G1/S transition required activation of glutamate receptors, given that NIH3T3 cells may express glutamate receptors, including NMDA glutamate receptors^28^, and that the concentrations of BMAA (1 and 3 mM) used in the present study were previously shown to induce excitotoxicity in neurons through NMDA glutamate receptor activation^7,19^. The excitotoxicity of BMAA is known to be strongly dependent on bicarbonate as a cofactor^7,19^. We therefore examined suppression of the G1/S transition using BMAA in Leibovitz’s L-15 medium, which does not contain bicarbonate^29^. The BrdU labeling index of BMAA-treated cells in L-15 medium was significantly lower than that of vehicle-treated control cells (Fig. 4a); however, the effects of BMAA on the G1/S transition appeared to be weaker in L-15 medium than in DMEM. We then examined whether the bicarbonate in L-15 medium enhanced suppression of the G1/S transition by BMAA. While the addition of 10 mM bicarbonate was previously shown to be sufficient for BMAA to induce excitotoxicity in neurons^7^, it did not enhance the BMAA-mediated suppression of the G1/S transition in L-15 medium. Instead, the bicarbonate alleviated the BMAA-mediated suppression of this transition (Fig. 4a). This result suggested that suppression of the G1/S transition by BMAA was independent of the activation of glutamate receptors. To confirm this hypothesis, we examined the G1/S transition in NIH3T3 cells treated with 1 mM glutamate, a concentration that is sufficient to induce nearly complete cell death in several neuroblastoma cell lines^20^. The BrdU labeling index of glutamate-treated cells (68.4%) was similar to that in control cells (61.2%) (Fig. 4b). Furthermore, we examined the effects of MK-801, a non-competitive NMDA glutamate receptor antagonist, on the suppression of the G1/S transition by BMAA because MK-801 treatment rescued neuronal cell death induced by BMAA in previous studies^1,3,18^. However, MK-801 did not counter the suppressive effects of BMAA on the G1/S transition in NIH3T3 cells (Fig. 4b).

**Figure 5:**
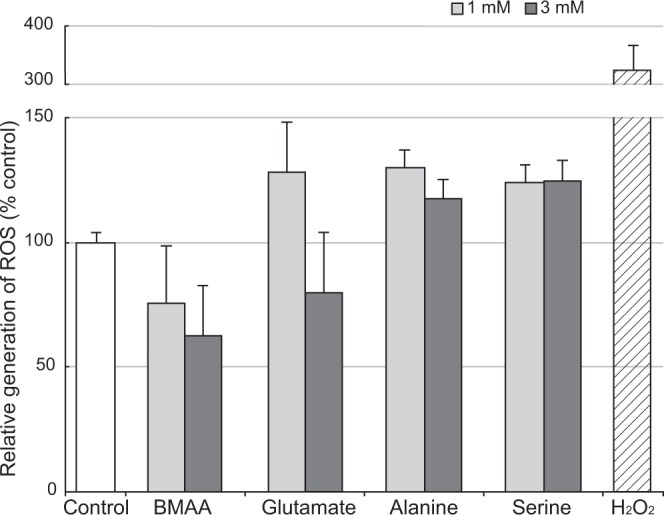
BMAA did not enhance ROS production in NIH3T3 cells. ROS production in cells treated with BMAA, glutamate, alanine, serine, or hydrogen peroxide (H_2_O_2_, positive control) was measured using a DCFDA assay. Data were normalized to the vehicle control values (open bar). Error bars represent SEM, n = 5.

Our results from the BrdU labeling and flow cytometry experiments indicate that BMAA potently arrested cell cycle progression at the G1/S checkpoint without activating glutamate receptors. Although G1 arrest may be induced by various factors, we focused on the overproduction of ROS^30^, which has been repeatedly observed in BMAA-treated cells^1,3,4,18,21,25^. To examine ROS production, we performed a dichlorofluorescein oxidation assay; however, we did not observe any significant differences in the generation of ROS between BMAA-treated NIH3T3 cells and control cells (Fig. 5).

**Figure 6:**
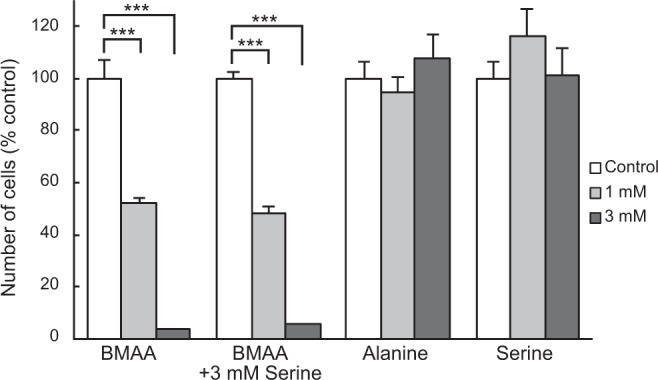
Co-incubation with serine did not prevent BMAA-mediated suppression of NIH3T3 cell proliferation. Cells were cultured for 96 h in medium containing BMAA, BMAA with serine, alanine, or serine. Data were normalized to vehicle control values (open bars). ***P < 0.001. Error bars represent SEM, n = 5.

A previous study^22^ demonstrated that co-incubation with serine and BMAA reduced the incorporation of BMAA into cellular proteins as well as its neurotoxicity^22^. We therefore investigated whether co-incubation with serine would alleviate the suppressed proliferation of BMAA-treated NIH3T3 cells. No significant differences were observed in the number of cells after incubation in medium containing BMAA with or without 3 mM serine for 96 h (Fig. 6).

## Discussion

BMAA is an excitatory neurotoxin^1,4–7,19^, and few studies have focused on its effects on non-neuronal cells. We revealed that BMAA potently suppressed the cell cycle progression of NIH3T3 cells, a fibroblast cell line, at the G1/S checkpoint. Apart from G1 arrest, we found no evidence of any detrimental effects on NIH3T3 cells following BMAA exposure, including cell membrane damage, apoptosis, or ROS overproduction, which have previously been reported in neuronal and OE cells^1,3,4,7,19,21,22,25^. Although we clearly demonstrated that BMAA induced G1 arrest in NIH3T3 cells, we cannot dismiss the possibility that it may induce cell cycle arrest at other checkpoints. A previous study demonstrated that BMAA was cytotoxic to OE cells^25^ and arrested cell cycle progression at the G2/M checkpoint^24^. BMAA may therefore also arrest the cell cycle of NIH3T3 cells at the G2/M checkpoint.

We found no evidence that glutamate receptor activation was involved in the G1 arrest of BMAA-treated NIH3T3 cells. Further, although bicarbonate has been shown to be essential for the excitotoxic effect of BMAA in neurons^7,19^, it was not required for BMAA-mediated suppression of the G1/S transition. Incubation with 1 mM glutamate, a concentration that is sufficient to induce cell death in several neuroblastoma cell lines, did not suppress the proliferation or G1/S transition of NIH3T3 cells. Furthermore, MK-801, which has been shown to attenuate neuronal cell death induced by BMAA, did not block the BMAA-mediated suppression of the G1/S transition^1,3,18^. These results indicate that BMAA induces G1 arrest of non-neuronal cells through glutamate receptor-independent mechanisms.

Arrest in the G1 phase of the cell cycle may occur due to various factors including the overproduction of ROS^30^, ER stress^31,32^, deficiency in nutrients and growth factors, and DNA damage. However, our results indicate that the overproduction of ROS is an unlikely cause of G1 arrest in BMAA-treated cells.

Previous studies have shown that BMAA is misincorporated into cellular proteins^21–23^, and autofluorescence indicating protein aggregation has been reported in BMAA-treated fibroblasts^22^. Intracellular protein aggregation may induce ER stress. BMAA reportedly causes ER stress in neuroblastoma cells via glutamate receptor-independent mechanisms^21^. Therefore, future studies should examine elevations in ER stress markers in BMAA-treated NIH3T3 cells.

Another possible cause of G1 arrest is DNA damage. BMAA induces DNA breaks in primary neurons, and excess ROS production has been suggested to cause BMAA-induced DNA damage^1^. However, we did not observe overproduction of ROS in BMAA-treated cells, suggesting that BMAA does not cause excessive DNA damage in NIH3T3 cells.

Nutrient deficiency is another possible cause of G1 arrest. BMAA is transported by L-type amino acid transporter 1 and possibly by other transporters^21^. BMAA may therefore be a competitive inhibitor of amino acid transporters and enzymes required for amino acid metabolism. The effects of BMAA on G1/S transition appeared to be weaker in L-15 medium than in normal medium (DMEM). L-15 medium is an amino acid-rich medium that contains alanine and asparagine (neither of which are present in DMEM), and five- to eight-fold higher concentrations of several amino acids, including arginine, glycine, histidine, and serine, than DMEM. These amino acids may compete with BMAA for transport or metabolism.

The addition of bicarbonate to L-15 medium unexpectedly alleviated the BMAA-mediated suppression of G1/S transition in NIH3T3 cells (Fig. 4a). Addition of bicarbonate to L-15 medium was previously shown to markedly enhance DNA synthesis and the proliferation of primary cultured hepatocyte cells^33,34^, an effect which is thought to be influenced by the high levels of amino acids in L-15 medium^34^. It is possible that the addition of bicarbonate to L-15 medium activates amino acid metabolism for DNA synthesis in NIH3T3 cells, and that this activated amino acid metabolism may mitigate the suppressive effect of BMAA on cell cycle progression.

Amino acid starvation or impaired amino acid metabolism may inhibit the nutrient-sensitive mammalian target of rapamycin (mTOR) pathway or activate the general control nonderepressible 2 (GCN2) pathway to protect cells against amino acid starvation^35^. Inhibition of the mTOR pathway suppresses cell cycle progression at the G1 phase^36,37^, and activation of the GCN2 pathway also leads to G1 arrest^38,39^. Further studies are needed to examine whether these pathways are involved in G1 arrest induced by BMAA.

A previous study^22^ showed that BMAA was misincorporated into proteins in place of serine, and that co-incubation with serine reduced the toxicity of BMAA in neuroblastoma cells. These findings prompted us to examine whether co-incubation with serine also attenuates the BMAA-mediated suppression of NIH3T3 cell proliferation; however, such attenuation was not observed. The finding that incubation in amino acid-rich L-15 medium appeared to mitigate the suppression of the G1/S transition in BMAA-treated cells, as described above, suggests that amino acids other than serine may compete with BMAA for transport or metabolism in NIH3T3 cells. Alternatively, the ratio of the concentration of serine to BMAA (1:1 and 3:1) used in the present study may have been insufficient for alleviation of the suppressed proliferation of BMAA-treated cells given that the concentration of serine used in the previous study^22^ was ten-fold higher than that of BMAA.

The mechanisms by which BMAA-induced G1 arrest contributes to the pathogenesis of ALS/PDC is currently unknown because most neurons in the adult brain are post-mitotic. However, neurons may reinitiate cell cycle progression under pathological conditions such as ischemia-hypoxia, excitotoxicity, or neurodegenerative diseases^40,41^. Although neuronal cell cycle re-entry may precede apoptosis, neurons in neurodegenerative diseases may actively re-enter the cell cycle and survive for long periods as tetraploid neurons^41^. The BMAA-induced suppression of cell cycle progression may therefore inhibit the production of tetraploid neurons and accelerate neuronal loss in neurodegenerative diseases, including ALS/PDC.

A recent study reported that an injection of a sub-toxic dose of BMAA into the cerebrospinal fluid delayed the progression of ALS in an ALS mouse model, which was suggested to have occurred due to prevention of the down-regulation of Na^+^/Ca^2+^ exchanger 3 (NCX3)^42^. NCX3 has been reported to be involved in cell cycle progression^43^. The subcellular localization of NCX3 changes during the cell cycle: NCX3 is detected in the plasma membrane during the S or M phases, but is mainly observed in the ER membrane in the G0/G1 phase. Moreover, forced expression of genetically modified NCX3 transported into the ER membrane perturbed cell cycle progression at the G1/S checkpoint. Therefore, future studies should clarify whether BMAA affects the expression of NCX3 in NIH3T3 cells.

In conclusion, we found that BMAA suppressed cell cycle progression in the mouse fibroblast cell line, NIH3T3, at least at the G1/S checkpoint, without activating glutamate receptors. As non-neuronal cell lines can easily be analyzed for metabolic changes and functional alterations, the present results provide a new perspective to understanding the mechanisms by which BMAA affects cellular function.

## Materials and Methods

All amino acids used in the present study were the L-form. BMAA (Sigma-Aldrich) was dissolved in 0.1 M phosphate buffer (pH 7.3) to 50 mM before being added to culture media. Since the excitotoxicity of BMAA is dependent on bicarbonate^7,19^, we used BMAA solution that was neutralized for sodium bicarbonate (50 mM BMAA, 50 mM phosphate buffer, and 100 mM sodium bicarbonate, pH 7.5) in some experiments.

NIH3T3 cells were maintained in Dulbecco’s modified Eagle’s medium (DMEM) containing 10% fetal bovine serum (FBS) (Gibco) in 5% CO_2_ at 37 °C.

### Treatment of culture cells with BMAA

NIH3T3 and HEK293T cells were seeded on 12- and 24-well culture plates, respectively, at a density of 1.2 × 10^4^ cells/well. This seeding density for NIH3T3 cells avoids contact inhibition. After cells were precultured for 16 h in DMEM containing 10% FBS (DMEM-FBS), medium was changed to DMEM-FBS containing BMAA (1 or 3 mM) for 48 h. To examine the effects of co-incubation with serine on the BMAA-induced suppression of NIH3T3 cell proliferation, cells were cultured in DMEM-FBS containing BMAA with 3 mM serine for 96 h after the preculture. Cells were then dissociated with 0.25% trypsin in phosphate-buffered saline (PBS) containing 0.02% ethylenediaminetetraacetic acid (EDTA). The number of cells was counted under a phase microscope using a hemocytometer. In a given experiment, all cell counts were performed by the same operator.

### LDH assay

To evaluate the cellular toxicity of BMAA, we examined the amount of LDH in culture medium released from membrane-damaged cells using a Cytotoxicity LDH Assay Kit-WST (DOJINDO Laboratories, Kumamoto, Japan). NIH3T3 cells were seeded on a 96-well culture plate according to the manufacturer’s protocol. After preculture for 16 h, medium was changed to DMEM-FBS containing BMAA, amino acids, or vehicle, and cells were cultured for 48 h prior to the LDH assay. Culture supernatants (100 µL) were transferred to another 96-well plate, and the substrate solution for the LDH assay was added to each well. After incubating for 30 min, LDH activity was assessed by measuring the absorbance at 490 nm. The amount of LDH released was expressed as a ratio of the control, in which all cells were lysed with 1.5% Triton X-100. The results obtained were normalized to the total protein content in each well to correct for well-to-well variations. Protein content was measured using a BCA protein assay kit (Thermo Fisher Scientific) after residual cells were solubilized with 1% sodium lauryl sulfate.

### ROS measurement

Oxidative stress was assayed by measuring dichlorofluorescein oxidation according to a previously reported method with modifications^3,18,44^. In this experiment, we used DMEM without phenol red to avoid interference with fluorescent measurements. After cells were cultured in DMEM-FBS for 16 h, they were incubated in medium containing BMAA or amino acids for 3 h in the presence of 10 μM 5(6)-carboxy-2′,7′-dichlorodihydrofluorescein diacetate (carboxy-H_2_DCFDA, Setareh Biotech, USA). Cells were then rinsed three times with DMEM, and fluorescence from the oxidation product of carboxy-H_2_DCFDA was measured using a fluorescent plate reader (EnSpire, PerkinElmer) with a 485-nm excitation filter and 538-nm emission filter. Background fluorescence (no carboxy-H_2_DCFDA added) was subtracted and data were presented as a % of the mean value calculated from control cultures (carboxy-H_2_DCFDA added, no treatment with BMAA).

### Analysis of BrdU incorporation

Approximately 1.2 × 10^4^ cells were seeded onto a coverslip in each well of a 12-well culture plate and preincubated for 16 h in DMEM-FBS. Medium was then changed to DMEM-FBS containing BMAA (1 or 3 mM) or alanine for the control. After 46 h, cells were pulse-labeled with 10 μM BrdU for 2 h. All experiments were performed in triplicate.

To examine G1/S progression, we synchronized cells at the G1 phase by incubating in DMEM containing 0.5% FBS for 48 h. Cells were then dissociated and seeded onto coverslips in a 12-well plate at 2 × 10^4^ cells/well. After the incubation in DMEM containing 10% FBS with BMAA, glutamate, alanine, 5 μM cytosine arabinoside, or vehicle for 16 h, cells were pulse-labeled with 10 µM BrdU for 2 h. To examine the effects of bicarbonate on the suppression of the G1/S transition by BMAA, we used Leibovitz’s L-15 medium instead of DMEM in air at 37 °C. Cells in L-15 medium containing 10 mM sodium bicarbonate were incubated in 2.1% CO_2_ to maintain the pH of the medium. Furthermore, the effects of NMDA receptor blockade on the suppression of the G1/S transition by BMAA were investigated using (+)-MK-801 maleate in combination with BMAA.

After incorporation of BrdU, cells were fixed in a mixture of methanol and acetic acid (3:1) for 30 min. After rinses with PBS containing 0.3% Triton X-100 (PBST) and PBS, fixed cells on coverslips were air-dried and denatured in 0.07 M NaOH for 2 min. After rinses with PBS, cells were incubated with blocking reagent (Blocking One; Nacalai Tesque, Japan) containing 5% normal goat serum (Invitrogen) for 1 h. Cells were then incubated with a mouse anti-BrdU antibody (dilution 1:1000, clone BU-33, Sigma-Aldrich) diluted with PBST containing 5% blocking reagent at 4 °C overnight. After rinses with PBST and PBS, cells were incubated with Alexa 488-labeled goat anti-mouse IgG (Thermo Fischer Scientific) diluted 1:500 in PBST containing 5% blocking reagent at room temperature for 1 h. After rinses with PBST and PBS, cells were counterstained with 4′,6-diamidino-2-phenylindole (DAPI, 1 µg/mL) diluted in PBS for 3 min. After several rinses with PBS, coverslips were mounted onto slide glasses with mounting medium. Cells were examined under a fluorescent microscope (Eclipse E800, Nikon) using a 20x objective, and images (at least five fields per culture) were randomly captured using a digital camera system (DXM1200F, Nikon). We then counted the number of DAPI-stained cells and BrdU-labeled cells on a liquid crystal monitor equipped with a digital camera. The BrdU-labeling index was calculated as the ratio of BrdU-positive nuclei to DAPI-stained nuclei. In each culture, we examined at least 50 DAPI-stained nuclei.

### Active caspase-3 immunostaining

Approximately 1.2 × 10^4^ cells were seeded onto a coverslip in each well of 12-well culture plates and preincubated for 16 h in DMEM-FBS. Medium was then changed to DMEM-FBS containing 1 mM BMAA, 1 mM alanine, or 50 μM etoposide, an inducer of apoptosis in NIH3T3 cells^45^, and the cells were cultured for 48 h.

Cells were fixed in 4% paraformaldehyde in 0.1 M phosphate buffer for 30 min. After rinses with PBST and PBS, cells were incubated with blocking reagent containing 5% normal goat serum for 1 h. Cells were then incubated with a rabbit anti-active caspase-3 antibody (dilution 1:500, clone C92–605, BD Biosciences) diluted with PBST containing 5% blocking reagent at 4 °C overnight. After rinses with PBST and PBS, cells were incubated with Alexa 488-labeled goat anti-rabbit IgG (Thermo Fisher Scientific) diluted 1:1000 in PBST containing 5% blocking reagent at room temperature for 1 h. After rinses with PBST and PBS, cells were counterstained with DAPI for 3 min. After several rinses with PBS, coverslips were mounted onto slide glasses with mounting medium. Cells were examined under a fluorescent microscope and images were captured by a digital camera. At least 500 DAPI-stained cells were examined per sample.

### Flow cytometry

NIH3T3 cells were synchronized at the G1 phase by incubating in low serum medium. Synchronized cells were dissociated and seeded at 2.8 × 10^5^ cells/10-cm dish. After incubating in DMEM-FBS containing 1 mM BMAA or vehicle for 22 h, cells were harvested via trypsinization and centrifuged at 200 × *g* for 5 min. Cells were resuspended and incubated in propidium iodide (PI)-staining solution containing 50 μg/mL PI, 0.25 mg/mL RNase A, 0.2% NP-40, 250 mM sucrose, and 5% DMSO in 4 mM sodium citrate buffer (pH 7.6) at 4 °C for 30 min following an incubation at 37 °C for 15 min to digest RNA. The fluorescence signal from 10,000 cells was analyzed using a flow cytometer (BD FACSVerse, BD Biosciences).

### Statistical analysis

All data, except those from the BrdU incorporation experiment, were examined using one-way analysis of variance (ANOVA) followed by the Tukey-Kramer HSD test. Data from the BrdU incorporation experiment were examined using repeated ANOVA followed by the Tukey-Kramer HSD test. All analyses were performed using JMP Pro 12 (SAS Institute).

## Data Availability

All data generated or analyzed during this study are included in this published article.
